# Engineering out the risk for infection with urinary catheters.

**DOI:** 10.3201/eid0702.010240

**Published:** 2001

**Authors:** D G Maki, P A Tambyah

**Affiliations:** University of Wisconsin Medical School, Madison, Wisconsin, USA. dgmaki@facstaff.wisc.edu

## Abstract

Catheter-associated urinary tract infection (CAUTI) is the most common nosocomial infection. Each year, more than 1 million patients in U.S. acute-care hospitals and extended-care facilities acquire such an infection; the risk with short-term catheterization is 5% per day. CAUTI is the second most common cause of nosocomial bloodstream infection, and studies suggest that patients with CAUTI have an increased institutional death rate, unrelated to the development of urosepsis. Novel urinary catheters impregnated with nitrofurazone or minocycline and rifampin or coated with a silver alloy-hydrogel exhibit antiinfective surface activity that significantly reduces the risk of CAUTI for short-term catheterizations not exceeding 2-3 weeks.


### Engineering Out the Risk for Infection with Urinary Catheters

**Dennis G. Maki* and Paul A. Tambyah†**
*University of Wisconsin Medical School, Madison, Wisconsin, USA, and
†National University of Singapore Medical School, Singapore

Catheter-associated urinary tract infection (CAUTI) is the most common nosocomial infection. Each year, more than 1 million patients in U.S. acute-care hospitals and extended-care facilities acquire such an infection; the risk with short-term catheterization is 5% per day. CAUTI is the second most common cause of nosocomial bloodstream infection, and studies suggest that patients with CAUTI have an increased institutional death rate, unrelated to the development of urosepsis. Novel urinary catheters impregnated with nitrofurazone or minocycline and rifampin or coated with a silver alloy-hydrogel exhibit antiinfective surface activity that significantly reduces the risk of CAUTI for short-term catheterizations not exceeding 2-3 weeks.

Each year, urinary catheters are inserted in more than 5 million patients in acute-care hospitals and extended-care facilities. Catheter-associated urinary tract infection (CAUTI) is the most common nosocomial infection in hospitals and nursing homes, comprising >40% of all institutionally acquired infections (1-4). Nosocomial bacteriuria or candiduria develops in up to 25% of patients requiring a urinary catheter for $\geq$ 7 days, with a daily risk of 5% (5-7). CAUTI is the second most common cause of nosocomial bloodstream infection (8-10), and studies by Platt et al. (11) and Kunin et al. (12) suggest that nosocomial CAUTIs are associated with substantially increased institutional death rates, unrelated to the occurrence of urosepsis. Although most CAUTIs are asymptomatic (13), rarely extend hospitalization, and add only $500 to $1,000 to the direct costs of acute-care hospitalization (14), asymptomatic infections commonly precipitate unnecessary antimicrobial-drug therapy. CAUTIs comprise perhaps the largest institutional reservoir of nosocomial antibiotic-resistant pathogens (5-10,15), the most important of which are multidrug-resistant Enterobacteriaceae other than *Escherichia coli*, such as *Klebsiella, Enterobacter, Proteus,* and *Citrobacter*; *Pseudomonas aeruginosa*; enterococci and staphylococci; and *Candida* spp. (Table 1).

Table 1. Microbial pathogens causing nosocomial catheter-associated urinary tract infections in U.S. acute-care hospitals, 1990-92 (15)

| Pathogens | Hospitalwide (% of total) | Intensive care units (% of total) |
|---|---|---|
| *Escherichia coli* | 26 | 18 |
| Enterococci | 16 | 13 |
| *Pseudomonas aeruginosa* | 12 | 11 |
| *Klebsiella* and *Enterobacter* spp. | 12 | 13 |
| *Candida* spp. | 9 | 25 |

Address for correspondence: Dennis G. Maki, H4/574 University of Wisconsin Hospital and Clinics, Madison, WI 53792, USA; fax: 608-231-3896; e-mail: dgmaki@facstaff.wisc.edu

### Pathogenesis

Excluding rare hematogenously derived pyelonephritis, caused almost exclusively by *Staphylococcus aureus*, most microorganisms causing endemic CAUTI derive from the patient's own colonic and perineal flora or from the hands of health-care personnel during catheter insertion or manipulation of the collection system. Organisms gain access in one of two ways (Figure 1). Extraluminal contamination may occur early, by direct inoculation when the catheter is inserted, or later, by organisms ascending from the perineum by capillary action in the thin mucous film contiguous to the external catheter surface. Intraluminal contamination occurs by reflux of microorganisms gaining access to the catheter lumen from failure of closed drainage or contamination of urine in the collection bag.

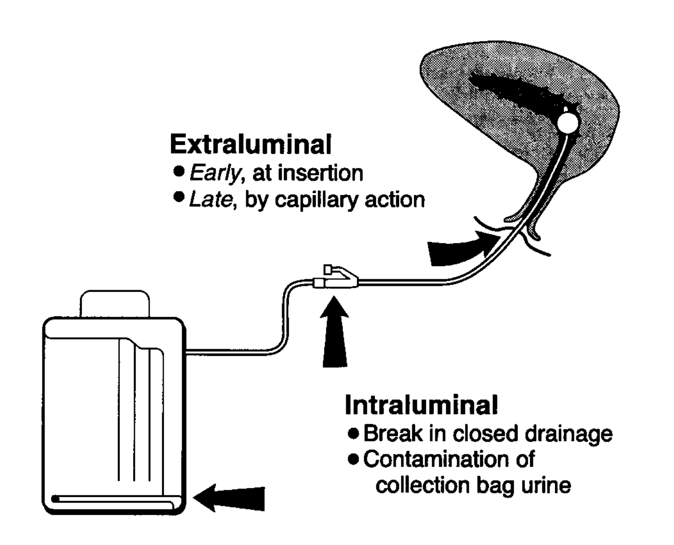

Figure 1. Routes of entry of uropathogens to catheterized urinary tract.

Recent studies suggest that CAUTIs most frequently stem from microorganisms gaining access to the bladder extraluminally, but both routes are important (Table 2) (16). Some studies suggest that the extraluminal route may be of greater relative importance in women because of the short urethra and its close proximity to the anus (17). Investigators have found that antecedent heavy periurethral cutaneous colonization is an important risk factor for CAUTI in both men and women (17,18).

Table 2. Mechanisms of catheter-associated urinary tract infection, based on a prospective study of 1,497 newly catheterized patients who had 235 new-onset infections (16)

| | Organisms causing CAUTI[a] | | | |
|---|---|---|---|---|
| Mechanism of CAUTI | Gram-positive cocci (n=44) | Yeasts (n=34) | Gram-negative bacilli (n=37) | Overall (n=115) |
| Extraluminal | 79% | 69% | 54% | 66% |
| Intraluminal | 21% | 31% | 46% | 34% |

[a]Percentages refer to organisms in which the mechanism of infection could be determined. For comparison of gram-positive cocci and yeasts vs. gram-negative bacilli, $p = 0.007$.
CAUTI = catheter-associated urinary tract infection.

Most infected urinary catheters are covered by a thick biofilm containing the infecting microorganisms embedded in a matrix of host proteins and microbial exoglycocalyx (Figure 2). A biofilm forms intraluminally, extraluminally, or both ways, usually advancing in a retrograde fashion (19). The role of the biofilm in the pathogenesis of CAUTI has not been established. However, antiinfective-impregnated and silver-hydrogel catheters (20-26), which inhibit adherence of microorganisms to the catheter surface, significantly reduce the risk of CAUTI, particularly infections caused by gram-positive organisms or yeasts, which are most likely to be acquired extraluminally from the periurethral flora (16). These data suggest that microbial adherence to the catheter surface is important in the pathogenesis of many, but not all, CAUTIs. Infections in which the biofilm does not play a pathogenetic role are probably caused by mass transport of intraluminal contaminants into the bladder by retrograde reflux of microbe-laden urine when a catheter or collection system is moved or manipulated (Figure 1, Table 2).

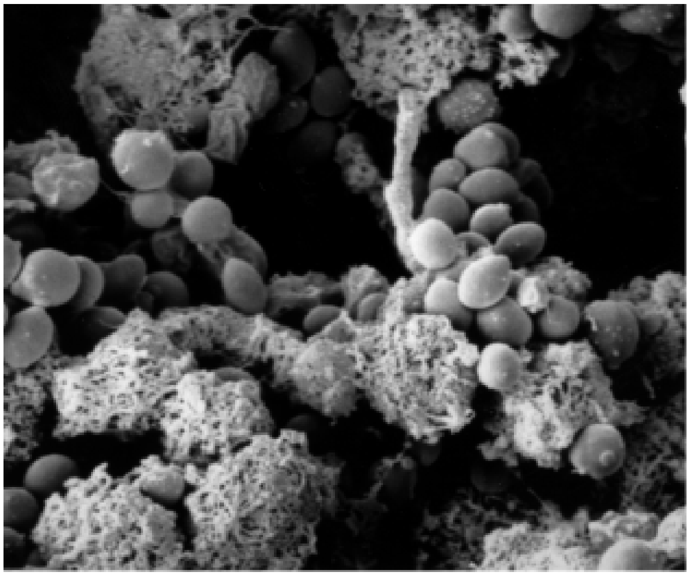

Figure 2. Scanning electron micrograph of an infected catheter showing dense and complex biofilm on the extraluminal surface. Urine culture at catheter removal yielded *Candida albicans* $10^4$ CFU/mL and *C. glabrata* $10^4$ CFU/mL (X 5000).

A prospective study in which catheterized patients were cultured daily by a technique capable of detecting very low-level bacteriuria, as low as 1 CFU/mL (7), showed that isolation of any microorganisms from an intraluminal specimen, even 3-4 CFU/mL, is highly predictive of CAUTI. If intercurrent antimicrobial therapy is not given, the level of bacteriuria or candiduria almost uniformly increases to $>10^5$ within 24-48 hours (Figure 3), demonstrating the vulnerability of the catheterized urinary tract to infection once any

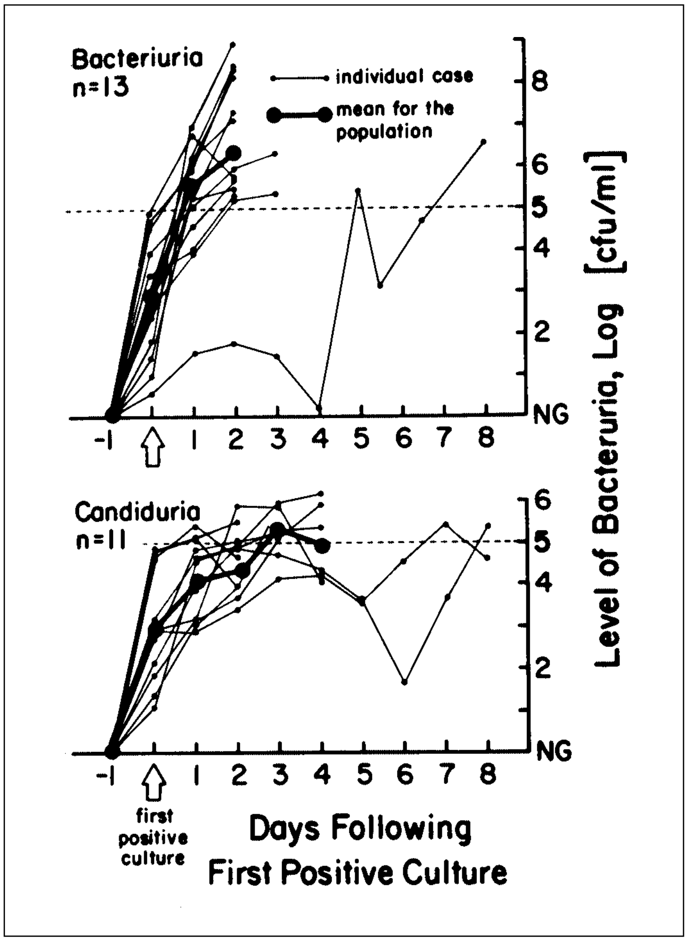

Figure 3. Rate of progression of bacteriuria and candiduria in 25 catheterized patients once any microorganisms were detectable in urine culture. Once organisms appeared in urine, low-level bacteriuria progressed very rapidly to levels $>10^5$ organisms per milliliter in 12 of the 14 cases within 2 days. Candiduria progressed less rapidly: in 9 of 11 cases, a concentration of $>10^5$ organisms per milliter was reached within 3 days (7).

microorganisms gain access to the lumen of the catheter and the bladder. The very heavy use of systemic antimicrobial drugs in catheterized patients, which has been found in most studies (5-13), probably keeps the rate of CAUTI considerably lower than it would be otherwise, but unfortunately selects for the resistant organisms that produce most nosocomial CAUTIs (Table 1).

### Definition of CAUTI

Most clinicians use a clean-voided specimen showing $>10^5$ CFU/mL as the criterion for "significant" bacteriuria (i.e., true infection) for noncatheterized patients (4). However, once any microorganisms are identified in urine from a patient's indwelling catheter, unless suppressive antimicrobial-drug therapy is being given or started, progression to concentrations $>10^5$ CFU/mL occurs predictably and rapidly, usually within 72 hours (Figure 3) (7). Thus, most authorities consider concentrations $>10^2$ or $10^3$ CFU/mL, in urine collected with a needle from the sampling port of the catheter, to be indicative of true CAUTI. This concentration can be reproducibly detected in the laboratory, and this definition is useful for therapeutic decisions and epidemiologic research (1-7).

### Risk Factors for CAUTI

Large, prospective studies in which catheterized patients were cultured daily and which used multivariable techniques of statistical analysis identified risk factors independently predictive of increased risk for CAUTI (27-30; Table 3). Females have a substantially higher risk than males (relative risk [RR] 2.5-3.7), and patients with other active sites of infection (RR 2.3-2.4) or a major preexisting chronic condition (such as diabetes [RR 2.2-2.3], malnutrition [RR 2.4], or renal insufficiency [RR 2.1-2.6]) also are at higher risk. Inserting the catheter outside the operating room (RR 2.0-5.3) or late in hospitalization (RR 2.6-8.6), presence of a ureteral stent (RR 2.5), or using the catheter to measure urine output (RR 2.0) further increase the risk.

The most important, potentially modifiable risk factor, identified in every study, is prolonged catheterization, beyond 6 days (RR 5.1-6.8); by the 30th day of catheterization, infection is near-universal. A large, prospective study monitored compliance on a daily basis with seven recommended precepts for catheter care, including closed drainage, dependent drainage including proper position of the drainage tubing and collection bag, and protection of the drainage port; the only violation predictive of an increased risk of CAUTI was improper position of the drainage tube, above the level of the bladder or sagging below the level of the collection bag (RR 1.9) (27).

Table 3. Risk factors for catheter-associated urinary tract infection, based on prospective studies and use of multivariable statistical modeling (27-30)

| Factor | Relative risk |
|---|---|
| Prolonged catheterization >6 days | 5.1-6.8 |
| Female gender | 2.5-3.7 |
| Catheter insertion outside operating room | 2.0-5.3 |
| Urology service | 2.0-4.0 |
| Other active sites of infection | 2.3-2.4 |
| Diabetes | 2.2-2.3 |
| Malnutrition | 2.4 |
| Azotemia (creatinine >2.0 mg/dL | 2.1-2.6 |
| Ureteral stent | 2.5 |
| Monitoring of urine output | 2.0 |
| Drainage tube below level of bladder and above collection bag | 1.9 |
| Antimicrobial-drug therapy | 0.1-0.4 |

Antimicrobial-drug therapy has been shown to be protective against CAUTI for short-term catheterizations (RR 0.001-0.4) but clearly selects for infection caused by multidrug-resistant microorganisms, such as *P. aeruginosa*, and other resistant gram-negative bacilli, enterococci, and yeasts (Table 1) (1-10,15).

### Guidelines for Preventing CAUTI

Several catheter-care practices are universally recommended to prevent or at least delay the onset of CAUTI: avoid unnecessary catheterizations; consider a condom or suprapubic catheter; have a trained professional insert the catheter aseptically; remove the catheter as soon as no longer needed; maintain uncompromising closed drainage; ensure dependent drainage; minimize manipulations of the system; and separate catherized patients (1-4). However, few of these practices have been proven to be effective by randomized controlled trials.

### Avoid Unnecessary Catheterizations

Use of indwelling urethral catheters should be limited to patients requiring relief of anatomic or physiologic outlet obstruction; patients undergoing surgical repair of the genitourinary tract (to facilitate healing); critically ill or postoperative patients who need their urinary output accurately measured; and debilitated, paralyzed, or comatose patients (to prevent skin breakdown and infected pressure ulcers). When no longer needed, the catheter should be promptly removed (31).

### Consider Alternatives to Urethral Catheterization

Suprapubic catheterization is more comfortable and acceptable to the patient and may be associated with a lower incidence of CAUTI (32). For incontinent males who do not have bladder outlet obstruction, condom drainage, while not free from nosocomial urinary tract infections, appears to be associated with a lower risk than indwelling urethral catheters (33).

### Insertion Using Aseptic Technique

Catheters should be inserted by trained health-care professionals using aseptic technique, including sterile gloves, a fenestrated sterile drape, and an effective cutaneous antiseptic, such as 10% povidone-iodine or 1% to 2% aqueous chlorhexidine.

### Closed Drainage

After a catheter is inserted, uncompromising maintenance of closed drainage is of the highest priority and can keep the overall risk of CAUTI <25% for up to 2 weeks of catheterization (5,6).

### Ensure Dependent Drainage

The collection tubing and bag should always remain below the level of the patient's bladder, but the drainage tubing should always be above the level of the collection bag.

In one large prospective study, this was the only catheter-care violation associated with a significantly increased risk of CAUTI (RR 1.9) (27).

### Urine Collection

The catheter and the drainage system should be manipulated as little as possible, and urine output should be monitored hourly only when clearly indicated by the patient's condition.

### Other Practices

If feasible, separating catheterized patients geographically on a patient-care unit may reduce the risk of cross-infection with multidrug-resistant nosocomial organisms such as *Serratia, Klebsiella, Pseudomonas,* and *Enterobacter* (34).

Systemic antimicrobial prophylaxis with trimethoprim-sulfamethoxazole, methenamine mandelate or, especially, a fluoroquinolone, can reduce the risk of CAUTI for short-term catheterizations (35). Although use of antimicrobials in this way may reduce the rate of CAUTI, infections that do occur are far more likely to be caused by antibiotic-resistant bacteria and yeasts (1-10). Since most CAUTIs are asymptomatic and do not result in urosepsis (13), it is difficult to justify antimicrobial therapy of asymptomatic bacteriuria other than for granulocytopenic or other severely immunocompromised patients, patients scheduled for urologic surgery, pregnant women, patients with *Serratia* CAUTI, or patients about to have their catheter removed. The societal benefits of antibiotic prophylaxis in immunocompetent catheterized patients to prevent largely asymptomatic CAUTIs are dubious.

### Novel Technology

Technologic innovations to prevent nosocomial infection are most likely to be most effective if they are based on a clear understanding of the pathogenesis and epidemiology of the infection (36). Novel technologies must be designed to block CAUTI by either the extraluminal or intraluminal routes or both (Figure 1). Technologic innovations have been proposed and evaluated during the past 25 years but have not proven conclusively beneficial (1-5). Among these innovations are using antiinfective lubricants when inserting the catheter; soaking the catheter in an antiinfective antimicrobial-drug solution before insertion; regular metal cleansing or periodically applying antiinfective creams or ointments to metals; continuously irrigating the catheterized bladder with an antiinfective solution through a triple-lumen catheter; or periodically instilling an antiinfective solution into the collection bag (Table 4). Bladder irrigation with antimicrobial-drug solutions has not only shown no benefit for prevention but has been associated with a strikingly increased proportion of CAUTIs caused by microorganisms resistant to the drugs in the irrigating solution (37).

Given the widely accepted importance of closed catheter drainage, efforts have been made to seal the connection between the catheter and collection tubing. An initial trial with a novel catheter showed a modest benefit and suggested a reduction in hospital deaths (38); however, follow-up studies have not demonstrated a reduction in CAUTI with a sealed catheter-collecting tube junction (39,40).

Medicated catheters, which reduce adherence of microorganisms to the catheter surface, may confer the greatest benefit for preventing CAUTI. Two catheters impregnated with antiinfective solutions have been studied in randomized trials, one impregnated with the urinary antiseptic nitrofurazone (20) and the other with a new broad-spectrum antimicrobial-drug combination, minocycline and rifampin (21). Both catheters showed a significant reduction in bacterial CAUTIs; however, the studies were small, and selection of antimicrobial-drug resistant uropathogens was not satisfactorily resolved.

Table 4. Studies of novel technologies for preventing catheter-associated urinary tract infection

| Technologic innovation (ref) | Risk reduction in randomized trials |
|---|---|
| Antiinfective lubricant (2) | Unproven |
| Sealed catheter-collection tubing junctions (38-40) | Unproven |
| Antireflux valves (2) | Unproven |
| Continuous irrigation of bladder with antiinfective solution (2,37) | Unproven |
| Instillation of antiinfective into collection bag (2) | Unproven |
| Antiinfective catheter material | |
| Antimicrobial drug-impregnated | |
| Nitrofurazone (20) | 0.7 (0.3[a]) |
| Minocycline-rifampin (21) | 0.4 |
| Silver oxide (29,30,42) | Unproven |
| Silver-hydrogel (22-25,26,42) | 0.2-0.7 |

CAUTI = catheter-associated urinary tract infection.
[a]For bacterial CAUTI.

The universal presence of a biofilm on the surface of an infected catheter (19) (Figure 2) has prompted hope that coating the catheter surface with an antiseptic, such as a silver compound, might reduce the risk for CAUTI. However, silver oxide-coated catheters, which had been initially reported to show promise, did not show efficacy when studied in large, well-controlled trials (29,30). In one of the trials, male patients with the coated catheter who did not receive systemic antibiotics had a paradoxical and inexplicably increased risk for CAUTI (30).

A silver-hydrogel catheter has been developed that inhibits adherence of microorganisms to the catheter surface in vitro; tested microorganisms include resistant enterococci, staphylococci, Enterobacteriaceae, *P. aeruginosa*, and yeasts (41). Small comparative but nonblinded trials have shown this product prevents CAUTI (22-25,42) (Figure 4). In a recent, large, double-blinded trial in 850 patients (26), the silver-hydrogel catheter reduced the incidence of CAUTI 26% (25.7 vs. 15.4 per 100 catheters, RR 0.74, p =0.04) (27). The greatest benefit was preventing infections caused by gram-positive organisms, enterococci and staphylococci (RR 0.45, p <0.001), and *Candida* (RR 0.80), microorganisms that usually gain access to the bladder extraluminally (16). The catheter conferred no protection against CAUTIs with gram-negative bacilli, which most often gain access intraluminally (16). Use of the silver-hydrogel catheter was not associated with an increased incidence of infections caused by antibiotic-resistant bacteria or *Candida*, and in vitro susceptibility testing of isolates from both treatment groups showed no infections caused by silver-resistant microorganisms. Cost-utility analysis indicates that use of this catheter could bring substantial cost savings to health-care institutions (Table 5).

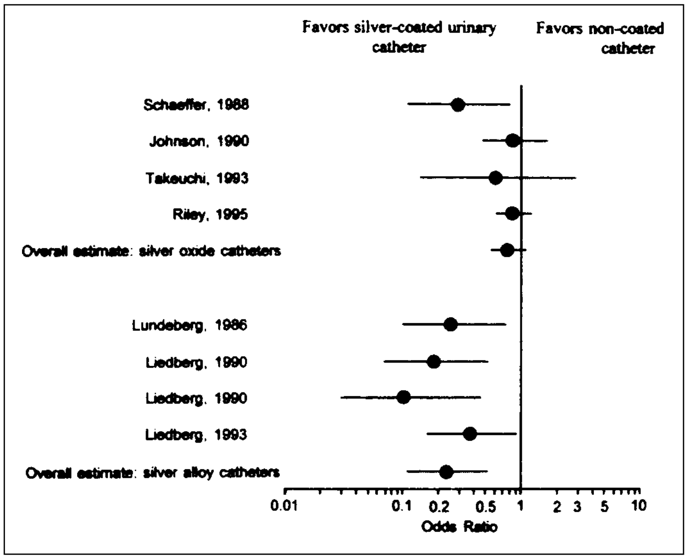

Figure 4. Meta-analysis of published prospective randomized trials of silver oxide and silver alloy-hydrogel catheters. Data suggest that silver-hydrogel catheters can substantially reduce the risk for CAUTI (42).

Table 5. Cost-benefit evaluation (restricted to direct hospital costs) of the silver-hydrogel catheter

| Assumptions of analysis | |
|---|---|
| Proportion of CAUTIs diagnosed clinically | 65% |
| Cost of each diagnosed CAUTI | ~$1000[a] |
| Added acquisition cost of a silver-hydrogel catheter | ~$5 |
| Incremental hospital costs, per 100 catheters: | |
| Using standard urinary catheters (26 CAUTIs, 17 diagnosed) | $17,000 |
| Using silver-hydrogel catheters (15 CAUTIs, 10 diagnosed) | $10,000 |
| Added cost of catheters | $500[b] |
| Total costs | $10,500 |
| Potential savings per 100 catheters | $6,500 |

[a]Based on studies showing that a diagnosed nosocomial CAUTI adds approximately $1,000 to direct costs of hospitalization (14); CAUTI = catheter-associated urinary tract infection.
[b]Cost of preventing a CAUTI: approximately $71.

### The Future

The first major advance for preventing CAUTI since the wide-scale adoption of closed drainage 35 years ago is the development of catheters with antiinfective surfaces. These advances should not be considered the final answer, however. Other technologies that should be pursued include new, more potent antiinfective materials; microbe-impervious antireflux valves; urethral stents; conformable (collapsible) urethral catheters; and vaccines for enteric gram-negative bacilli and staphylococci. Antiseptics are far more likely than antibacterials to confer greater resistance to surface colonization and not to select for infection with antimicrobial-drug resistant bacteria or yeasts (43). New surface technologies that release far greater quantities of ionic silver or other antiinfective agents into the aqueous environment contiguous to the catheter surface might even prevent CAUTIs caused by intraluminal contaminants.

In uncontrolled trials, urethral stents have provided a less-invasive alternative to catheter drainage for men with outlet obstruction caused by prostatic hypertrophy or cancer (44). A conformable catheter, with a collapsible intraurethral segment that may cause less trauma to the urethra, has been developed but has not been tested clinically and is not commercially available. These and other alternatives to the rigid urethral catheter, such as a condom catheter for female patients (45), need to be evaluated in controlled, randomized trials.

The greatest hope for a major reduction in CAUTI and indeed all nosocomial infections is likely to be vaccines against important nosocomial multidrug-resistant pathogens, such as the enteric gram-negative bacilli and staphylococci.

Dr. Maki is professor of medicine and head of the Section of Infectious Diseases at the University of Wisconsin Medical School and hospital epidemiologist at University of Wisconsin Hospitals and Clinics. He has had a long interest in the pathogenesis, epidemiology, and prevention of nosocomial infections, particularly those caused by catheters or other implanted medical devices.

Dr. Tambyah, formerly a clinical and research fellow in infectious diseases at the University of Wisconsin Medical School, is assistant professor of medicine and consultant infectious disease physician at the University of Singapore School of Medicine and hospital epidemiologist at the National University Hospital of Singapore.

### References

1. Stamm WE. Catheter-associated urinary tract infections: Epidemiology, pathogenesis, and prevention. Am J Med 1991;91(Suppl 3B):65S-71S.
2. Burke JP, Riley DK. Nosocomial urinary tract infection. In: Mayhall CG, editor. Hospital epidemiology and infection control. Baltimore: Williams and Wilkins; 1996. p. 139-53.
3. Warren JW. Catheter-associated urinary tract infections. Infect Dis Clin North Am 1997;11:609-22.
4. Kunin CM. Care of the urinary catheter. In: Urinary tract infections: detection, prevention and management. Fifth ed. Baltimore: Williams and Wilkins; 1997. p. 227-99.
5. Kunin CM, McCormack RC. Prevention of catheter-induced urinary-tract infections by sterile closed drainage. N Engl J Med 1966;274:1155-61.
6. Garibaldi RA, Mooney BR, Epstein BJ, Britt MR. An evaluation of daily bacteriologic monitoring to identify preventable episodes of catheter associated UTI. Infect Control 1982;3:466-70.
7. Stark RP, Maki DG. Bacteriuria in the catheterized patient. N Engl J Med 1984;311:560-4.
8. Maki DG. Nosocomial bacteremia. An epidemiologic overview. Am J Med 1981;70:719-32.
9. Krieger JN, Kaiser DIL, Wenzel RP. Urinary tract etiology of bloodstream infections in hospitalized patients. J Infect Dis 1983;148:57-62.
10. Bryan CS, Reynolds KL. Hospital-acquired bacteremic urinary tract infection: epidemiology and outcome. J Urol 1984,132:494-8.
11. Platt R, Polk BF, Murdock B, Rosner B. Mortality associated with nosocomial urinary-tract infection. N Engl J Med 1982;307:637-41.
12. Kunin CM, Douthitt S, Dancing J, Anderson J, Moeschberger M. The association between the use of urinary catheters and morbidity and mortality among elderly patients in nursing homes. Am J Epidemiol 1992;135:291-301.

13. Tambyah PA, Maki DG. Catheter-associated urinary tract infection is rarely symptomatic: a prospective study of 1497 catheterized patients. Arch Intern Med 2000;160:678-82.
14. Patton JP, Nash DB, Abrutyn E. Urinary tract infection: economic considerations. Med Clin North Am 1991;75:495-513.
15. Jarvis WR, Martone WJ. Predominant pathogens in hospital infections. J Antimicrob Chemother 1992;29:19-24.
16. Tambyah PA, Halvorson, K, Maki DG. A prospective study of the pathogenesis of catheter-associated urinary tract infection. Mayo Clin Proc 1999;74:131-6.
17. Daifuku R, Stamm WE. Association of rectal and urethral colonization with urinary tract infection in patients with indwelling catheters. JAMA 1984;252:2028-30.
18. Garibaldi RA, Burke JP, Britt MR, Miller MA, Smith CB. Metal colonization and catheter-associated bacteriuria. N Engl J Med 1980;303:316-18.
19. Nickel JC, Costerton JW, McLean RJ, Olson M. Bacterial biofilms: influence on the pathogenesis, diagnosis and treatment of urinary tract infections. J Antimicrob Chemother 1994;33(Suppl A):31-41.
20. Maki DG, Knasinski V, Halvorson KT, Tambyah PA, Holcomb RG. A prospective, randomized, investigator-blinded trial of a novel nitrofurazone-impregnated urinary catheter [abstract M49]. Infect Control Hosp Epidemiol 1997;18(Suppl):50.
21. Darouiche RO, Smith A, Hanna H, Dhabuwala CB, Steiner MS, Babaian RJ, et al. Efficacy of antimicrobial-impregnated bladder catheters in reducing catheter-associated bacteriuria: a prospective, randomized multicenter clinical trial. Urology 1999;54:976-81.
22. Lundeberg T. Prevention of catheter-associated urinary tract infections by use of silver-impregnated catheters [letter]. Lancet 1986;1:1031.
23. Liedberg H, Lundeberg T. Silver alloy coated catheters reduce catheter-associated bacteriuria. Br J Urol 1990;65:379-81.
24. Liedberg H, Lundeberg T, Ekman P. Refinements in the coating of urethral catheters reduce the incidence of catheter-associated bacteriuria. An experimental and clinical study. Eur Urol 1990;17:236-40.
25. Liedberg H, Lundeberg T. Prospective study of incidence of urinary tract infection in patients catheterized with bard hydrogel and silver-coated catheters or bard hydrogel-coated catheters [abstract 405A]. J Urol 1993;149.
26. Maki DG, Knasinski V, Halvorson K, Tambyah PA. A novel silver-hydrogel impregnated indwelling catheter reduces CAUTIs: a prospective double-blind trial [abstract]. In: Programs and abstracts of the Society for Healthcare Epidemiology in America Annual Meeting; April 5-7, 1998; Orlando, Florida.
27. Maki DG, Knasinski V, Tambyah PA. Risk factors for catheter-associated urinary tract infection: a prospective study showing the minimal effects of catheter care violations on the risk of CAUTI [abstract]. Infect Control Hosp Epidemiol 2000;21:165.
28. Platt R, Polk BF, Murdock B, Rosner B. Risk factors for nosocomial urinary tract infection. Am J Epidemiol 1986;124:977-85.
29. Johnson JR, Roberts PL, Olsen RJ, Moyer KA, Stamm WE. Prevention of catheter-associated urinary tract infection with a silver oxide-coated urinary catheter: Clinical and microbiologic correlates. J Infect Dis 1990;162:1145-50.
30. Riley DK, Classen DC, Stevens LE, Burke JP. A large randomized clinical trial of a silver-impregnated urinary catheter: Lack of efficacy and staphylococcal superinfection. Am J Med 1995;98:349-56.
31. Rabkin DG, Stifelman MD, Birkhoff J, Richardson KA, Cohen D, Nowygrod R, et al. Early catheter removal decreases incidence of urinary tract infections in renal transplant recipients. Transplant Proc 1998;30:4314-16.
32. Shapiro J, Hoffmann J, Jersky J. A comparison of suprapubic and transurethral drainage for postoperative urinary retention in general surgical patients. Acta Chirurgica Scandinavia 1982;148:323-7.
33. Warren JW. Urethral catheters, condom catheters, and nosocomial urinary tract infections. Infect Control Hosp Epidemiol 1996;17:212-14.
34. Maki DG, Hennekens C, Bennet J. Prevention of catheter-associated urinary tract infection. JAMA 1972;221:1270-1.
35. van der Wall E, Verkooyen RP, Mintjes-de-Groot J, Oostinga J, Van Dijk A, Hustius WN, et al. Prophylactic ciprofloxacin for catheter-associated urinary tract infection. Lancet 1992;339:946-51.
36. Maki DG. Risk factors for nosocomial infection in intensive care. "Devices vs nature" and goals for the next decade. Arch Intern Med 1989;149:30-5.
37. Warren JW, Platt R, Thomas RJ, Rosner B, Kass EH. Antibiotic irrigation and catheter-associated urinary tract infections. N Engl J Med 1978;299;570-3.
38. Platt R, Polk BF, Murdock B, Rosner B. Reduction of mortality associated with nosocomial urinary tract infection. Lancet 1983;1:893-6.
39. Huth TS, Burke JP, Larsen RA, Classen DC, Stevens LE. Clinical trial of junction seals for the prevention of urinary catheter-associated bacteriuria. Arch Intern Med 1992;152:807-12.
40. Classen DC, Larsen RA, Burke JP, Stevens LE. Prevention of catheter-associated bacteriuria: clinical trial of methods to block three known pathways of infection. Am J Infect Control 1990;19:136-42.
41. Gabriel MM, Sawant AD, Simmons RB, Hearn DG. Effects of silver on adherence of bacteria to urinary catheters: in vitro studies. Curr Microbiol 1995;30:1722.
42. Saint S, Elmore JG, Sullivan SD, Emerson SS, Koepsell TD. The efficacy of silver alloy-coated urinary catheters in preventing urinary tract infection: a meta-analysis. Am J Med 1998;105:236-4.
43. Maki DG, Stolz SM, Wheeler S, Mermel LA. Prevention of central venous catheter related bloodstream infection by use of an antiseptic-impregnated catheter. Ann Intern Med 1997;127:257-66.
44. Nissenkorn I. The intraurethral catheter-three years of experience. Eur Urol 1993;24:27-30.
45. Johnson DE, O'Reilly JL, Warren JW. Clinical evaluation of an external urine collection device for nonambulatory incontinent women. J Urol 1989;141:535-7.
